# Age-associated sperm DNA methylation patterns do not directly persist trans-generationally

**DOI:** 10.1186/s13072-019-0323-4

**Published:** 2019-12-19

**Authors:** Timothy G. Jenkins, Emma R. James, Kenneth I. Aston, Albert Salas-Huetos, Alexander W. Pastuszak, Ken R. Smith, Heidi A. Hanson, James M. Hotaling, Douglas T. Carrell

**Affiliations:** 1 Department of Physiology and Developmental Biology, Brigham Young University Provo, Life Sciences Building 4005, Provo, UT 84602 USA; 2 Andrology and IVF Laboratories, University of Utah School of Medicine, Salt Lake City, UT USA; 3 Department of Surgery (Urology Division), University of Utah School of Medicine, Salt Lake City, UT USA; 4 Department of Genetics, University of Utah School of Medicine, Salt Lake City, UT USA; 5 Department of Population Sciences, University of Utah School of Medicine, Salt Lake City, UT USA

## Abstract

**Background:**

The impact of aging on the sperm methylome is well understood. However, the direct, subsequent impact on offspring and the role of altered sperm DNA methylation alterations in this process remain poorly understood. The well-defined impact of aging on sperm DNA methylation represents an excellent opportunity to trace the direct, transgenerational transmission of these signals.

**Results:**

We utilized the Illumina MethylationEPIC array to analyze the sperm of 16 patients with older (> 40 years of age) paternal grandfathers (‘old grand paternal age’ patients; OGPA) and 16 patients with younger (< 25 years of age) grandfathers (‘young grand paternal age’ patients; YGPA) identified through the Subfertility Health Assisted Reproduction and the Environment (SHARE) cohort to investigate differences in DNA methylation. No differentially methylated regions were identified between the OGPA and YGPA groups. Further, when assessing only the sites previously shown to be altered by age, no statistically significant differences between OGPA and YGPA were identified. This was true even despite the lower bar for significance after removing multiple comparison correction in a targeted approach. Interestingly though, in an analysis of the 140 loci known to have decreased methylation with age, the majority (~ 72%) had lower methylation in OGPA compared to YGPA though the differences were extremely small (~ 1.5%).

**Conclusions:**

This study suggests that the robust and consistent age-associated methylation alterations seen in human sperm are ‘reset’ during large-scale epigenetic reprograming processes and are not directly inherited trans-generationally (over two generations). An extremely small trend was present between the YGPA and OGPA groups that resemble the aging pattern in older sperm. However, this trend was not significant and was so small that, if real, is almost certainly biologically inert.

## Background

Transgenerational and intergenerational inheritance are fascinating, but poorly understood, phenomena that are of great interest in many scientific disciplines including the basic and health sciences. The level of interest is due to the unique inheritance patterns observed, where a parent experiences reversible alterations to gametes that are capable of impacting offspring (intergenerational inheritance) and even grand offspring (transgenerational inheritance) phenotypes in a manner independent of genetic mutation. Despite the high level of interest, a great deal of controversy surrounds the process [1, 2], largely due to the fact that the precise mechanism(s) driving the observed patterns remain poorly elucidated [2].

A key roadblock in defining mechanisms that underlie these patterns of inheritance is the fact that they behave more like Lamarckian proposed heritability patterns as opposed to Mendelian mechanisms. Further, these patterns likely have the capacity to result in both protective and/or deleterious effects in the offspring [3–5]. In effect, the currently proposed mechanism suggests that the environment to which an adult is exposed can impact epigenetic signatures in the gametes which are, by definition, mitotically stable and thus have the potential to impact offspring phenotype. The precise signatures and nature of the alterations appear to be unique for each exposure type, adding to the difficulty in tracing these patterns over multiple generations.

Most prominent in the recent transgenerational and intergenerational inheritance literature are studies assessing the impacts of environment and other epigenetic modifiers on sperm that are believed to have the potential to impact offspring phenotype [6–9]. Indeed, based on early evidence from these studies, the identified environmentally driven alterations to the sperm appear to have the capacity to alter offspring phenotype, though the mechanism of action has largely remained elusive. Still, this research has yielded impressive findings suggesting that a father can contribute traits to the offspring beyond the DNA code alone. Such claims are in stark contrast to the previously held dogma that men contribute little beyond a DNA blueprint to the embryo and ultimately, the offspring.

A recent focus of the field has been the impact of age on sperm and subsequent offspring. This is so prominent that a term has been coined to describe the patterns identified, namely, “The Paternal Age Effect”. This term describes all of the impacts of advancing paternal age on offspring. Data from animal models, epidemiological studies, and human tissues suggest that not only can the age of the father impact offspring health, but also that there are potential mechanisms to further explore that may explain some of this effect. Epidemiological studies have shown that the offspring of older men have a higher incidence of various neuropsychiatric disorders (autism, schizophrenia, bipolar disorder, etc.) [10–13]. Some studies of autism even suggest that advanced grand paternal age (meaning the age at which the grandfather conceived the father of the child in question) is a potential risk factor for autism (a finding which meets the rigorous criteria associated with true transgenerational inheritance) [11]. Studies in mice have identified behavioral abnormalities consistent with neuropsychiatric disease symptoms in the offspring of older mice, with impacts persisting for multiple generations [13, 14]. It should be noted that some portion of this effect may be a result of mutations though it is unlikely the only causative factor.

In human studies, it has been shown that sperm have unique epigenetic alterations associated with age that are enriched at genes associated with bipolar disorder and schizophrenia [8]. Interestingly, this signature is so strong and consistent that we recently built a germ line age calculator using only these sites [15]. This predictive model is capable of using sperm DNA methylation signatures to predict, with a high degree of accuracy, the chronological age of the individual. Of further interest is the fact that the aging signal in sperm is far different from that of somatic cells and is, in fact, virtually opposite in terms of direction of change [15, 16]. While the mechanism still remains uncertain, it is clear that paternal age has a significant impact on the offspring and that sperm epigenetic signatures likely play a role.

To further explore the direct heritability of DNA methylation and its association with the mechanistic underpinnings of transgenerational inheritance, we analyzed the impact of grand paternal age on the sperm methylome. This study design was employed to assess direct transgenerational inheritance for a number of reasons. First, aging is among the most profound and predictable modifiers of sperm epigenetic profiles [8, 15, 17]. Second, transgenerational inheritance by definition requires that a mark is passed on over at least two generations. Lastly, we have access to two unique resources including a robust tissue bank with sperm samples from thousands of men, and the Utah Population Database (UPDB) which links genealogical and medical records for many generations allowing us to determine the age of an individual’s family members and ancestors over multiple generations. The UPDB was linked to our semen analysis bio bank to create the SHARE cohort (Subfertility Assisted Reproduction and the Environment) which now comprises multigenerational pedigree data on half the cohort, demographic, medical, semen analysis, fertility and fecundity data on over 1.5 million people some of which have tissue stored in our research bio bank [18].

Thus, we utilized the unique tools at our disposal to perform an assessment of the heritability of significantly modified epigenetic signatures (as consistently occur with age) from a grandparent to their grand offspring, which provides a powerful means to address the question of direct, transgenerational epigenetic inheritance of DNA methylation patterns.

## Results

### Study group

**Fig. 1:**
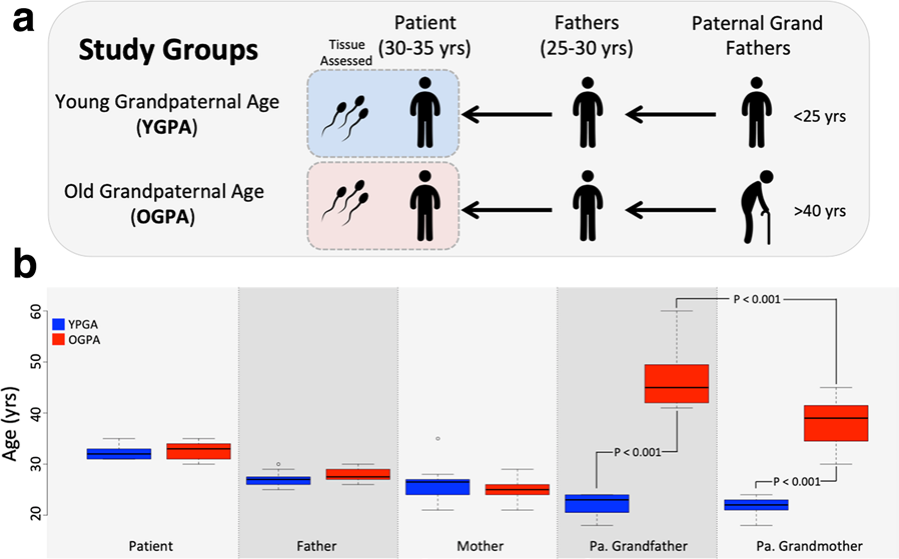
Diagram depicting study design where patients are divided into two groups based on grand paternal age (**a**). Boxplots showing the ages of the patients as well as their parents and grandparents in the two study groups assessed (**b**)

The study group comprised 32 previously stored sperm samples from two distinct study groups (2 × *n* = 16). In both groups, every effort was made to isolate the variable of grand paternal age to fully assess transgenerational epigenetic inheritance patterns in humans. Inclusion in each group of the study required that the individual who collected the sample (patient) was between 30 and 35 years of age at the time of sample collection and that their fathers were between 25 and 30 years old at the time of conception. To be included in the ‘Old Grand Paternal Age’ (OGPA) arm of the study, the patient’s paternal grandfather needed to be older than 40 years of age at the time of conception of the patient’s father. To be included in the ‘Young Grand Paternal Age’ (YGPA) arm of the study the patient’s paternal grandfather needed to be less than 25 years of age at the time of conception of the patient’s father. Figure 1 illustrates the study design (A) and provides the data regarding ages of patients, their parents, and the paternal grandparents (B).

### Differential methylation analysis

We utilized Illumina’s 850k (EPIC) array to generate DNA methylation data. To assess whether any inherent DNA methylation differences existed between the two groups, we performed differential methylation analysis by means, and with computational tools, commonly employed in our lab [8]. Specifically, we performed differential analysis at multiple levels including *point analysis* (single CpG level), *regional analysis*, and *global analysis*.

#### Point analysis

Using the software package minfi [19], we assessed single CpG level differential methylation between OGPA and YGPA. Following multiple comparison correction, no CpG sites were identified to be differentially methylated between the two groups.

#### Regional analysis

Regional differential methylation analysis was performed in the OGPA and YGPA groups using minfi and the methylation array scanner application in the USEQ software package. We did not identify any differentially methylated regions using multiple techniques with various thresholds of significance.

#### Global analysis

We assessed differences between the OGPA and YGPA groups in the global level of methylation as calculated by an overall average of all fraction methylation values (beta values) tiled on the array, within each individual. No significant difference was identified.

### Germ line age calculation

**Fig. 2:**
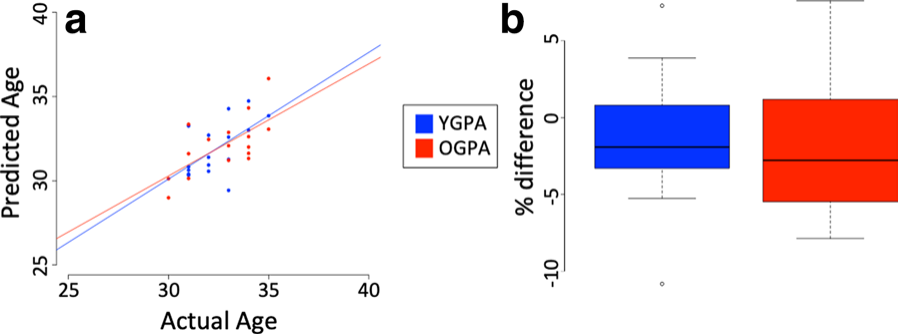
Scatter plot showing the results of the germ line age calculation on each of the study groups (**a**). Box plot showing the germ line age differential (the accuracy of the prediction of the germ line age calculator) in both study groups (**b**)

To determine whether the offspring of older grandfathers displayed accelerated aging patterns, we calculated each individual’s germ line age based on the recently published calculator [15]. This algorithm calculates a predicted age for an individual and is capable of detecting age acceleration or deceleration patterns in individuals whose germ line age is greater or less than their chronological age. The germ line age was successfully predicted for patients from both the OGPA and YGPA groups (Fig. 2a). However, no significant germ line age difference (GLAD) between the OGPA and YGPA groups was observed (Fig. 2b).

### Assessment of trends restricted to loci known to have a loss of methylation

**Fig. 3:**
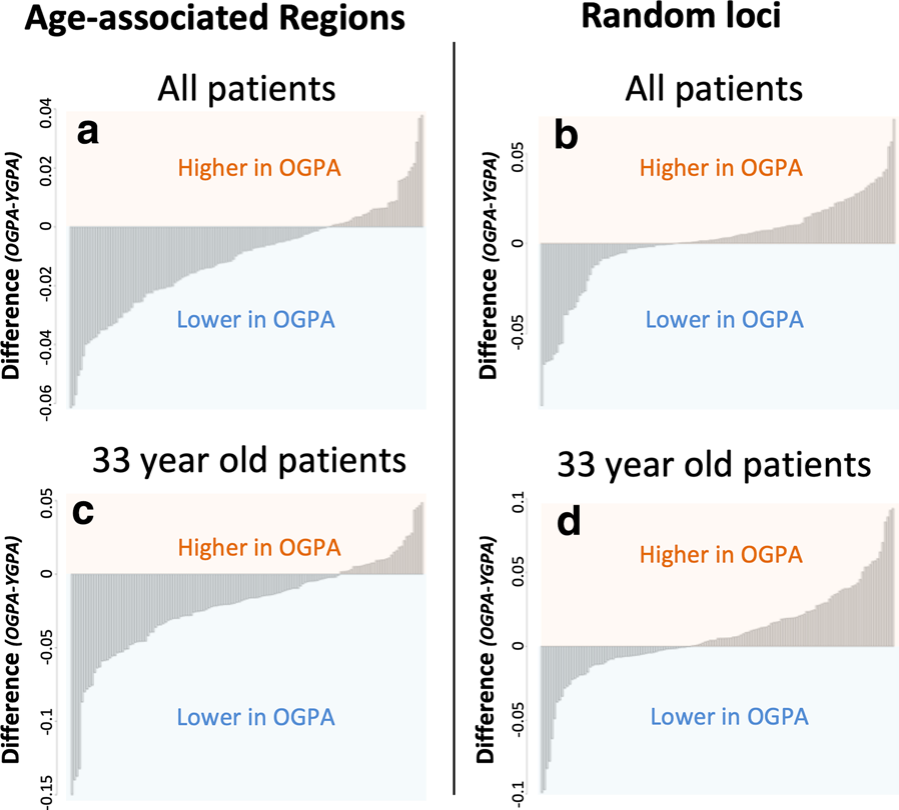
Histograms depicting the direction and magnitude of sperm DNA methylation difference between the OGPA group and YGPA groups at the 140 sites known to decrease with age in sperm (**a**) as well as at random loci covered on the array throughout the genome (**b**). Additional histogram only analyzing members of the OGPA and YGPA groups that are 33 years of age (**c**, **d**)

To more directly assess the heritability of alterations known to occur with age, we assessed more subtle methylation alteration at only the 140 regions identified previously that consistently display decreased methylation values with age. In addition to limiting the number of regions assessed, we also removed thresholds for magnitude of alterations. This analysis was designed to determine if the methylation signal was more frequently lower in the OGPA patients compared to the YGPA patients regardless of the magnitude of this effect. We assessed the average methylation values at the 140 age-associated regions in both the OGPA and YGPA groups. ~ 72% of the 140 sites assessed had, on average, lower methylation levels in OGPA patients compared to YGPA patients at the same age-associated regions (Fig. 3a).

### Assessment of trends over the entire genome

In a similar effort to assess only the 140 regions known to lose methylation with age, we also assessed random locations outside of these regions across the entire genome to determine if these loci follow similar trends to those described in previous work [8]. Specifically, previous studies have shown that there is a global gain of DNA methylation associated with increasing age outside of the regions described above. To accomplish this, 1000 sites were randomly selected throughout the genome from which an average fraction methylation value was generated for each site in both the OGPA and YGPA groups. In contrast to what we identified when assessing only the 140 regions that have decreased methylation with age, we found that ~ 64.4% of sites had higher methylation in OGPA patients when compared to YGPA patients (Fig. 3b).

### Validation in only 33-year-old patients

To confirm that the above findings were not a result of any small differences in age between the OGPA and YGPA patients, we assessed only the patients that were 33 years old in both the OGPA (n = 3) and YGPA (n = 4) groups. We first assessed the 140 regions of the genome that are known to have age-associated decreased methylation in the 33-year-old patient cohort. Similar to what was identified in the full data set, we found that ~ 75% of the regions assessed displayed lower methylation in the OGPA group compared to the YGPA group at these sites (Fig. 3c). Additionally, when we randomly selected loci across the genome in the 33-year-old cohort we found that only ~ 60% of loci had increased methylation in the OGPA group when compared to the YGPA group (Fig. 3d). These findings comport well with those identified in the entire study group.

## Discussion

The study of transgenerational and/or intergenerational epigenetic inheritance holds tremendous promise and implications, but lacks a fundamental understanding of mechanisms that drive the process. To address this, we designed our study to specifically assess the direct transmission of altered DNA methylation signatures in sperm over two generations. Keys to such an approach are identifying alterations that are common among all individuals, are easily quantifiable, and are located in discrete positions throughout the genome. The patterns of aging in the sperm epigenome meet these criteria perfectly. Sperm DNA methylation signatures are significantly altered in a very predictable manner in all men at distinct locations [8]. In fact, previous work from our lab successfully identified the regions of the sperm epigenome that are consistently altered (typically with a loss of methylation) as a result of aging and also shown that outside of these regions there tends to be higher methylation globally [8, 17]. To illustrate the consistency of these regional alterations, our lab has utilized these sites to build an aging calculator that powerfully and accurately can predict an individual’s chronological age based on the sperm DNA methylation signature alone [15]. Because the aging signal is so robust, it represents the ideal signal to attempt to trace over multiple generations.

One of the key issues in the transmission of epigenetic information from one generation to the next in humans is the massive epigenetic reprogramming that occurs following fertilization and during embryogenesis [20]. A hallmark of this process is the widespread and active erasure of DNA methylation marks in the paternal genome [21]. Such a process can clearly limit the ability to directly pass on DNA methylation signatures from the father to the offspring. However, this erasure is incomplete and thus DNA methylation signatures in certain areas of the genome including imprinted genes are capable of escaping this erasure and may be directly passed on to the epigenome of the offspring. Importantly, previous work has shown that even some sites that are not imprinted may become ‘imprinted-like’ and are capable of being passed down to offspring and even grand offspring as has been demonstrated in even very recent work [22].

We were unable to find the direct transgenerational inheritance patterns that have been shown elsewhere via the imprinted-like inheritance pattern. In fact, our analysis showed that methylation signatures in sperm that are present in older men had been ‘reset’ during embryonic reprograming. We first assessed this by performing differential methylation analysis across the entire array in addition to only the sites known to change with age in the sperm of grandsons of older grandfathers. We were unable to identify any significant differences. We secondly assessed whether the sperm from the OGPA group appeared to be older than the sperm from the YGPA group despite being, effectively, the same age using the newly developed germ line aging calculator. Similarly, no differences were identified between groups. Further, when testing for differential methylation by typical means across the entire array, no significant findings were identified. Thus, our findings suggest that aging signals are not directly passed from one generation to the next and are likely completely erased and reestablished in the early embryo.

Further analysis sought to identify any traces of what could be considered an aging-like signal in OGPA patients. We did identify an extremely modest and non-significant trend for OGPA patients to display a bias toward reduced methylation at the 140 age-associated regions known to have lower sperm DNA methylation in older men, and for global methylation changes to display a bias of increased methylation as has been shown to occur with advanced age [8, 17]. These effects were extremely subtle and as such, even if proven to be a real remnant of an aging signal would be, effectively, biologically inert.

Our findings demonstrate that the DNA methylation signature of aging in sperm is not passed on and maintained directly over two generations. This is likely due to the widespread erasure of paternal DNA methylation signatures through the epigenetic reprograming processes in the early embryo. If this is the case, it is unlikely that any of the sites impacted by aging would even be capable of direct transmission over a single generation (though this was not assessed in our study). This process is intuitive from an evolutionary perspective as it prevents DNA methylation signals altered over the lifespan of a father to accumulate over multiple generations. It is not difficult to imagine the potential negative implications of the compounding of this effect over multiple generations of older fathers. Despite the negative findings in our study, there has been a great deal of previous work that has shown the inheritance patterns of altered DNA methylation signatures in sperm over multiple generations [23–25]. In each case, both DNA methylation alterations and disease persisted over time. This suggests that inheritance may be unique to each exposure of epigenetic modifier. While age appears to be programmatically removed, exposure to environmental toxicants, at least those studied above, may be able to persist over time. More work is needed to understand the dynamic and complex nature of these inheritance patterns, where they exist, and why they may be different between different epigenetic modifiers.

There are some limitations to this work. One of the key issues to consider is the fact that we cannot fully isolate grand paternal age because grand paternal age is significantly associated with grand maternal age. Thus, grand maternal age is a confounder in our particular study design. While a similar increase in grand maternal age is an inherent issue with our outlined study design, it is unlikely to be a key driver of the specific patterns we are assessing in the offspring for a number of reasons. First, it is believed that the genesis of age-related epigenetic alterations is tightly correlated with cell division/proliferation [26]. Due to a lack of cell divisions, oocytes are unlikely to have strong DNA methylation signals of aging, although this has not been fully explored in humans. In contrast, the highly proliferative nature of sperm appears to generate significant (both in number and magnitude) age-associated epigenetic alterations. Second, our study focuses on the areas of the sperm genome known to be impacted by age that, because of the unique nature of sperm, are distinct from the patterns of aging in other cell types. Thus, it is more likely that impacts found in the offspring at these sites are the result of paternal and not maternal contributions.

## Conclusions

Taken together, our data demonstrate that the signals of aging in sperm are erased in the early embryo and not directly passed on trans-generationally in humans. However, we did identify a trend that some very subtle and likely biologically inert remnants of sperm aging be present over two generations. It is clear though that despite the fact that some remnants of aging may be detectable, these signals are not of high enough magnitude and/or consistency to result in any sort of biological consequences. This is supported by the fact that there was no significant increase in the germ line age of patients in the OGPA group compared to the YGPA group. The value of this finding is that it may offer some insight into the robust nature of epigenetic reprograming mechanisms in the embryo. A great deal more work is required to further elucidate these mechanisms and to determine if our observations offer any additional utility.

## Methods

### Study group

A total of 32 previously stored sperm samples were assessed in this study. The sperm were collected by standard protocols at the University of Utah, were mixed with a cryomedium (Test Yolk Buffer) and stored in liquid nitrogen prior to being thawed and assessed for this study.

These samples were from two distinct study groups (2 × *n* = 16). In both groups, every effort was made to isolate the variable of grand paternal age to fully assess transgenerational epigenetic inheritance patterns in humans. Semen and health parameters in our patient population are included in Table 1. Inclusion in each group of the study required that the individual who collected the sample (patient) was between 30 and 35 years of age at the time of sample collection and that their fathers were between 25 and 30 years old at the time of conception. To be included in the ‘Old Grand Paternal Age’ (OGPA) arm of the study the patient’s paternal grandfather needed to be older than 40 years of age at the time of conception of the patient’s father. To be included in the ‘Young Grand Paternal Age’ (YGPA) arm of the study the patient’s paternal grandfather needed to be less than 25 years of age at the time of conception of the patient’s father. The Utah population database was integral in identifying patients available in our tissue bank that met the criteria outlined above.

**Table 1:** Patient characteristics for those included in the study

	BMI (SE)	Concentration (SE)	Motility (SE)	Total count (SE)	Total motile (SE)	Percent motility (SE)	Viability (SE)
OGPA	25.10 (1.14)	69.32 (7.92)	60.78 (4.21)	215.34 (28.5)	110.09 (17.76)	48.43 (5.06)	60.33 (2.96)
YGPA	24.80 (1.83)	83.90 (15.91)	53.36 (5.04)	283.89 (56.58)	143.69 (29.61)	44.93 (4.55)	56.78 (3.99)
p value	0.8927	0.4187	0.2678	0.2879	0.3383	0.6107	0.486

Sperm DNA extraction, bisulfite conversion and array processing: all samples were thawed and prepared for DNA extraction. Prior to DNA extraction, we performed somatic cell lysis as is commonly used in our lab to remove any potentially contaminating somatic cells [8]. We observed the samples under a light microscope to ensure purity. Following somatic cell lysis, we subjected the samples to DNA extraction using the DNeasy column-based extraction kit (Qiagen; Germantown, MD) with a sperm-specific modification commonly employed in our lab [8]. Extracted DNA was then subject to bisulfite conversation with EZ DNA methylation kit (Zymo; Irvine, CA). A small portion of the bisulfite-converted DNA was used to perform a somatic cell contamination check designed to ensure that the extracted DNA is free of somatic cell DNA as we have previously described [27]. Following this, we submitted the bisulfite-converted DNA to the Genomics core lab at the University of Utah for array hybridization and processing.

### Data handling

Once the raw data were received from the core lab, we utilized the software package minfi to generate fraction methylation values (termed ‘beta values’ which are between 0 and 1; 0 being complete absence of methylation and 1 being completely methylated) while also performing SWAN normalization. These values were then used in all downstream differential methylation applications.

### Differential methylation analysis

To assess whether any inherent DNA methylation differences existed between the two groups, we performed differential methylation analysis by means, and with computational tools, commonly employed in our lab.

#### Point analysis

Using the software package minfi we assessed single CpG level differential methylation between OGPA and YGPA.

#### Regional analysis

Regional differential methylation analysis was performed for the OGPA and YGPA groups using minfi and the methylation array scanner (MAS) application in the USEQ software package.

#### Global analysis

We assessed differences between the OGPA and YGPA groups in the global level of methylation as is calculated by an overall average of all fraction methylation values (beta values) within each individual.

### Germ line age calculation

To determine whether the offspring of older grandfathers displayed accelerated aging patterns, we calculated each individual’s germ line age based on the recently published calculator. This algorithm calculates a predicted age for an individual and is capable of detecting age acceleration or deceleration patterns in individuals whose germ line age is greater or less than their chronological age. These patterns are described by the germ line age differential (GLAD), which is calculated by the equation: GLAD = (predicted age/actual age − 1) × 100.

### Assessment of trends restricted to loci known to have a loss of methylation

To more directly assess the heritability of alterations known to occur with age, we assessed more subtle methylation alteration at only the 140 regions identified in a previous publication that consistently display decreased methylation values with age. In addition to limiting the number of regions assessed, we also removed thresholds for magnitude of alterations. This analysis was designed to determine if the methylation signal was more frequently lower in the OGPA patients compared to the YGPA patients regardless of the magnitude of this effect.

### Assessment of trends over all sites tiled on the array

In a similar effort to assessing only the 140 regions known to lose methylation with age, we also assessed random locations outside of these regions across the entire array to determine if on average these loci follow similar trends to those described in previous work [8]. Specifically, previous studies have shown that there is a global gain of DNA methylation outside of the regions described above. To accomplish this, we randomly selected 1000 sites covered on the array from across the genome and generated average fraction methylation values for each site in both the OGPA and YGPA groups.

### Validation in only 33-year-old patients

To confirm that the above findings were not a result of any small differences in age between the OGPA and YGPA patients, we assessed only patients that were 33 years of age in both the OGPA (*n* = 3) and YGPA (*n* = 4) groups. We assessed the 140 regions of the genome that are known to have decreased methylation in the 33-year-old patient cohort and also assessed 1000 randomly selected loci for differences between the OGPA group and the YGPA group.

## Data Availability

Illumina 850k array data are available through Gene Expression Omnibus (GEO). **Accession number to access data will be added after data are fully uploaded.*
